# Identification of Rare Recurrent Copy Number Variants in High-Risk Autism Families and Their Prevalence in a Large ASD Population

**DOI:** 10.1371/journal.pone.0052239

**Published:** 2013-01-14

**Authors:** Nori Matsunami, Dexter Hadley, Charles H. Hensel, G. Bryce Christensen, Cecilia Kim, Edward Frackelton, Kelly Thomas, Renata Pellegrino da Silva, Jeff Stevens, Lisa Baird, Brith Otterud, Karen Ho, Tena Varvil, Tami Leppert, Christophe G. Lambert, Mark Leppert, Hakon Hakonarson

**Affiliations:** 1 Department of Human Genetics, University of Utah, Salt Lake City, Utah, United States of America; 2 Center for Applied Genomics, The Children's Hospital of Philadelphia, Philadelphia, Pennsylvania, United States of America; 3 Lineagen, Inc., Salt Lake City, Utah, United States of America; 4 Golden Helix, Inc., Bozeman, Montana, United States of America; 5 Department of Pediatrics, University of Pennsylvania School of Medicine, Philadelphia, Pennsylvania, United States of America; Emory University School Of Medicine, United States of America

## Abstract

Structural variation is thought to play a major etiological role in the development of autism spectrum disorders (ASDs), and numerous studies documenting the relevance of copy number variants (CNVs) in ASD have been published since 2006. To determine if large ASD families harbor high-impact CNVs that may have broader impact in the general ASD population, we used the Affymetrix genome-wide human SNP array 6.0 to identify 153 putative autism-specific CNVs present in 55 individuals with ASD from 9 multiplex ASD pedigrees. To evaluate the actual prevalence of these CNVs as well as 185 CNVs reportedly associated with ASD from published studies many of which are insufficiently powered, we designed a custom Illumina array and used it to interrogate these CNVs in 3,000 ASD cases and 6,000 controls. Additional single nucleotide variants (SNVs) on the array identified 25 CNVs that we did not detect in our family studies at the standard SNP array resolution. After molecular validation, our results demonstrated that 15 CNVs identified in high-risk ASD families also were found in two or more ASD cases with odds ratios greater than 2.0, strengthening their support as ASD risk variants. In addition, of the 25 CNVs identified using SNV probes on our custom array, 9 also had odds ratios greater than 2.0, suggesting that these CNVs also are ASD risk variants. Eighteen of the validated CNVs have not been reported previously in individuals with ASD and three have only been observed once. Finally, we confirmed the association of 31 of 185 published ASD-associated CNVs in our dataset with odds ratios greater than 2.0, suggesting they may be of clinical relevance in the evaluation of children with ASDs. Taken together, these data provide strong support for the existence and application of high-impact CNVs in the clinical genetic evaluation of children with ASD.

## Introduction

Twin studies [1]–[3], (reviewed in [4]), family studies [5]–[7], and reports of chromosomal aberrations in individuals with ASD (reviewed in [8]) all have strongly suggested a role for genes in the development of ASD. Although the magnitude of the genetic effect observed in ASD varies from study to study, it is clear that genetics plays a significant role.

While a number of genes associated with ASD susceptibility have been observed in multiple studies, variants in a single gene cannot explain more than a small percentage of cases. Indeed, recent estimates suggest that there may be nearly 400 genes or chromosomal regions involved in ASD predisposition [9]–[12].

In the past few years, a number of studies have identified both *de novo* and inherited structural variants, including CNVs, that are associated with ASD [13]–[23]. *De novo* CNVs may explain at least some of the “missing heritability” of ASD as understood to date. While it is clear that CNVs play an important role in susceptibility to ASD, it is also clear that the genetic penetrance of many of these CNVs is less than 100%. Although many of the duplications or deletions observed in children with ASD occur as *de novo* variants, duplications, for example on chromosome 16p11.2, often are inherited from an asymptomatic parent. Moreover, both deletions and duplications encompassing a portion of chromosome 16p11.2 have been associated with ASD [21], [24]–[26] and 16p11.2 gains have been associated with ADHD and schizophrenia [24], [27]–[29], indicating that the same genomic region can be involved in multiple developmental conditions. In addition, deletions on chromosome 7q11.23 are known to cause Williams syndrome and duplications of this same region have been observed and are thought to be causal in individuals with ASD [9], [11]. While individuals with Williams syndrome tend to be outgoing and social, individuals with ASD are socially withdrawn, suggesting that deletions and duplications in this region result in individuals on opposite sides of the behavioral spectrum.

Although numerous studies regarding the role of CNVs in ASD have been published in the research literature, the findings of these studies have not been fully utilized for clinical evaluation of children with ASD. This is likely due to the rarity of individual variants, the lack of probe coverage on clinical microarrays that permits detection of smaller variants, and the difficulty in understanding the relevant biology of some variants even when they are significantly associated with ASD. Despite this, published clinical guidelines suggest that microarray-based testing should be the first step in the genetic analysis of children with syndromic and non-syndromic ASD as well as other conditions of childhood development [30], and there is a wealth of information demonstrating its utility in large samples of children who have undergone such testing [25], [31].

In this work we describe our efforts to discover high-impact CNVs in high-risk ASD families in Utah and to assess their potential role in unrelated ASD cases. We interrogated these CNVs, as well as CNVs from multiple published sources [18], [32] in a large sample set of ASD cases and controls, to determine more precisely their potential disease relevance. To evaluate carefully these CNVs, we designed a custom Illumina iSelect array containing probes within and flanking CNV regions of interest. We used this custom array to obtain high-quality CNV results on 2,175 children with clinically diagnosed ASD and 5,801 children with normal development following removal of samples that did not meet our stringent quality control parameters. The results of this study identify multiple rare recurrent CNVs from high-risk ASD families that also confer risk in unrelated ASD cases and delineate the prevalence and impact of CNVs reported in the literature in a large case control study of ASDs.

## Results

### CNV discovery in Utah high risk autism pedigrees

Using CNAM (GoldenHelix Inc.) on Affymetrix Genome-Wide Human SNP array 6.0 data, we identified a total of 153 CNVs in subjects with autism in Utah families that were not found in any of our CEPH/UGRP control samples. This set included 131 novel CNVs and 22 CNVs present in the Autism Chromosomal Rearrangement Database [15]. Thirty-two autism-specific CNVs were detected in multiple (2 or more) autism subjects, and 121 CNVs were detected in only one person among the 55 autism subjects assayed. Of these 153 CNVs, 112 were copy number losses (deletions) and 41 were copy number gains (duplications). The average size of the CNVs from high-risk families was 91 kb. The genomic locations of these CNVs are shown in Table S2 in File S1.

### CNV regions on the custom array

To better understand the frequency of the CNVs identified in Utah ASD families in a broader ASD population, we created a custom Illumina iSelect array containing probes covering all 153 of the Utah CNVs described in Table S2 in File S1. CNV coordinates, copy number status, and probe content for each CNV are included. In addition, since the ultimate goal of this work is to understand the frequency and relevance of rare recurrent CNVs in the etiology of ASD, we included probes for 185 autism-associated CNVs identified in the literature [14]–[16], [18], [21], [32], [33] (Table S3 in File S1). The probe coverage for each literature CNV also is shown in Table S3 File S1. In total, 7134 probes, all selected from the Illumina 2.5 M array, were used for this study. As part of a separate study we also included 2,799 SNVs detected by next-generation sequencing of genes in regions of haplotype sharing among our high-risk ASD families and in published ASD candidate genes in these same individuals. Intensity data for these SNVs were used to identify additional CNVs that were not observed in our Utah high-risk ASD families (Table S4 in File S1). Following standard data QC steps (see supplemental results) this array was used to characterize which of these 363 CNVs were present in DNA from 2,175 children with autism and 5,801 age, gender, and ethnicity matched controls (Table 1). These 7976 samples were available for analysis following our strict quality control measures (File S2).

**Table 1:** Case and control samples used in this study.

	case		control	
	male	female	male	female
AGRE/AGP	1,517	626	0	0
CHOP	633	224	3,992	2,008
sub-total	2,150	850	3,992	2,008
grand-total	3,000		6,000	

### Analysis of CNVs on the iSelect array

**Figure 1:**
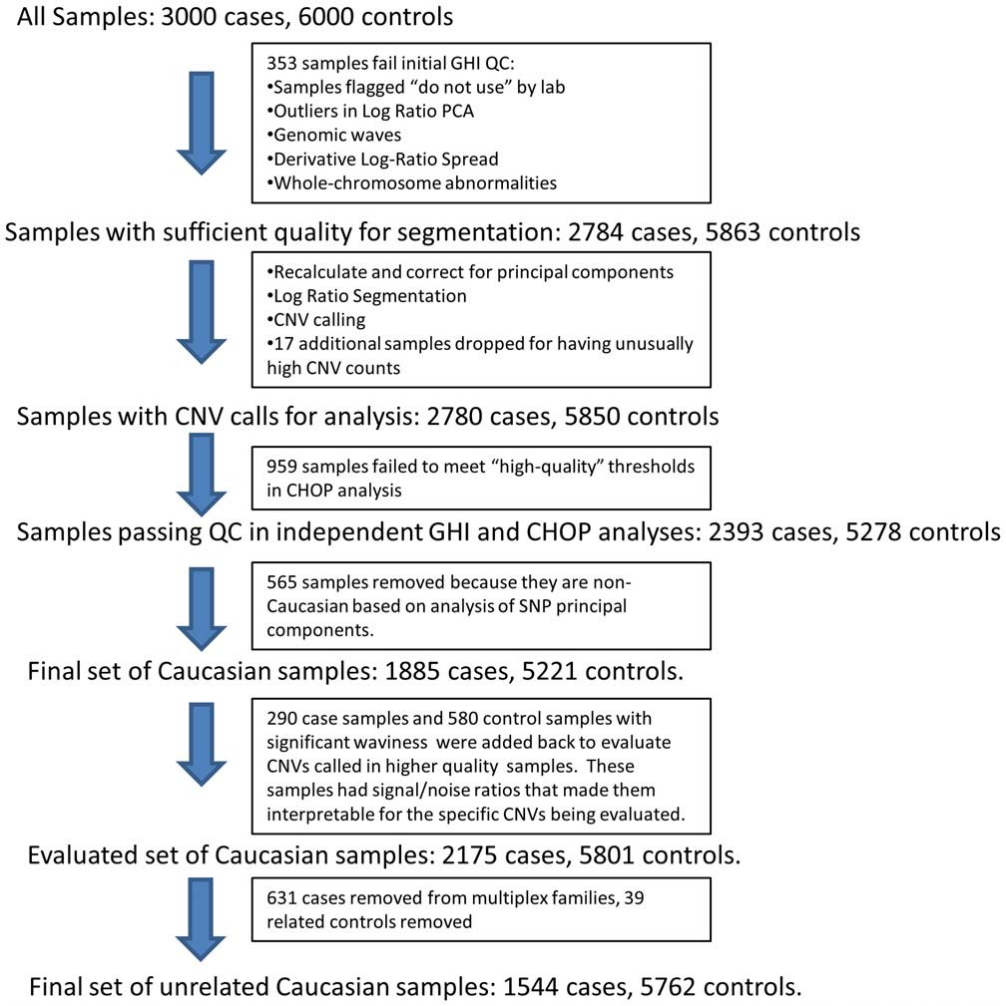
Workflow for CNV analysis for samples analyzed on the custom array. The same process was used for both CNAM and PennCNV analyses. All samples used for CNV analysis in this study had to meet the quality control measures described. Only unrelated cases and controls were used for the final statistical analysis.

The workflow for CNV analysis of the custom array data is shown in Figure 1. Following quality control analysis, including removal of samples that did not meet laboratory sample quality control measures, samples with excessive CNV calls, samples of uncertain ethnicity, and related samples, our final dataset included 1544 unrelated cases and 5762 unrelated controls. Because of the inherent noisiness of CNV analysis, we used two independent CNV calling algorithms, PennCNV [34] and CNAM (Golden Helix, Inc.), to increase our ability to detect CNVs. We identified 6,086 CNVs in cases and 14,387 CNVs in controls using PennCNV and 3,226 CNVs in cases and 8,234 CNVs in controls using CNAM. 1,537 CNVs from the 2175 cases including those from multiplex families (average 0.70 CNVs per individual) and 3,845 CNVs from the 5801 controls including related controls (average of 0.66 CNVs per individual) were called by both algorithms used for CNV detection.

**Figure 2:**
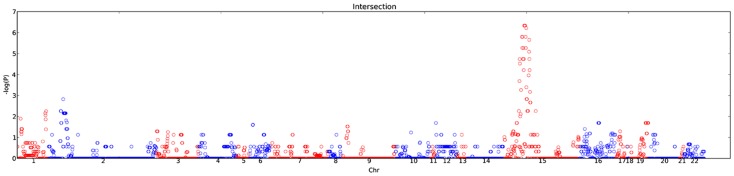
Manhattan plot of CNVs called both by PennCNV and CNAM. Association statistics across all regions covered on the Illumina custom array are shown. Since the array used was not a genome-wide array, the width of each chromosome on the plot is not proportional to the chromosome length. Adjacent chromosomes are displayed in alternating red and blue colors to aid in distinguishing them.

All CNV regions harboring CNVs shared among subjects were defined from PennCNV calls, CNAM calls and the PennCNV/CNAM intersecting calls and their significance of association was calculated across the genome (Figure 2). Of the 153 CNVs discovered in high-risk ASD families, 139 of them were seen in replication samples evaluated with the custom Illumina iSelect array. Seven of the CNVs not seen in this larger population study had poor probe coverage on the array either due to their small size or their genomic content, while the remainder that were not detected may represent false positive CNVs from our initial discovery work or may be rare CNVs that are private to the families or individuals in which they were identified.

### Molecular validation of CNV calls

We used TaqMan copy number assays to confirm the presence of CNVs in our population. A summary of the 195 TaqMan assays used is shown in Table S1 in File S1. Since our goal for this study was to understand the frequencies of these CNVs in a large case/control population, we chose to validate any CNVs that were likely to have clinical relevance. Our criteria for selection were as follows: 1) any CNV with an odds ratio > = 2.0; 2) any rare CNV seen in at least two cases. We chose these criteria for selecting CNVs to validate because our goal was to translate research CNV findings into potentially clinically useful markers. Since clinical testing of individuals with ASD is only performed on people who are symptomatic, CNVs with odds ratios <1.0 (CNVs that indicate lower than average risk of ASD) were not chosen for validation. Likewise, since CNVs with odds ratios > = 1 but < = 2 do are not of great diagnostic interest, we chose to validate only CNVs with odds ratios > = 2.0. By using these criteria, we included rare recurrent CNVs that may be etiologically important despite the lack of statistical significance in cases versus controls. For previously published CNVs we considered our custom Illumina iSelect array as an independent test of their validity. We assumed therefore that these CNVs did not require additional testing. Since some of the CNVs from The Children's Hospital of Philadelphia (CHOP) were not included in previous publications [18], [32], we selected all CHOP CNVs for molecular validation. For CNVs that met our selection criteria we assayed a maximum of six case samples that contained the CNV, giving priority to those samples called both by PennCNV and CNAM. Results of these TaqMan experiments are summarized in Table 2. Interestingly, many of the most common CNVs detected by the array were not validated by the TaqMan assays. For example, when we tested samples from a statistically significant CNV duplication on chromosome 7q36.1 that was detected only by PennCNV and not by CNAM, all samples tested were shown to have two copies rather than the anticipated three copies, suggesting that in this sample set at least some of the CNV duplications observed are not true positives. Conversely all but one of the CNVs observed on chromosome 15, whether in the Prader-Willi/Angelman syndrome region or located more distally on chromosome 15, were confirmed by TaqMan assays. Results of these validation experiments demonstrated that CNVs called both by PennCNV and CNAM were much more likely to be confirmed (97% of tested samples) than CNVs called by either PennCNV alone (24%) or CNAM alone (30%). This observation demonstrates the care that must be taken during the CNV discovery process to insure that only valid calls are selected for further analysis.

**Table 2:** Confirmation of CNV calls by quantitative PCR.

TaqMan CNV Validation Status	Utah Family CNVs	Utah Sequence SNP CNVs	Literature CNVs	Total
PASS	24 (2 overlap with Lit. CNV)	15	25	64
FAIL	9	9	5	23
NoCall	0	1	0	1

A summary of the PCR validation result is shown. Sequence SNP CNVs were discovered in this work using SNVs present on this array for sequence variant confirmation in the same cohort.

False negative results also are possible with our microarray studies. However, the controls that we used for TaqMan assays were selected from our control sample set because they lacked CNV calls for any of the regions being evaluated. In none of these samples did the TaqMan results indicate the presence of any of the CNVs being validated, so no false negative results were detected. These data suggest that false negative results are not a common problem in this study.

### CNVs from high-risk Utah families

One hundred thirty-nine of the 153 CNVs identified in high-risk ASD families were observed in case and/or control samples in this large dataset. Of these, 33 were present in two or more cases and had odds ratios greater than 2 and thus were selected for molecular confirmation. Following TaqMan validation, fifteen of the thirty-three CNVs were validated (Table 3). Of the 15 validated CNVs identified in high-risk families, 4 were shown to be inherited CNVs while three were *de novo* CNVs in the discovery families. The remainder were of undetermined origin, in most cases due to lack of information for one or both parents. A CNV that was validated in some samples but not in others, for example if a CNV was validated in all calls made by both PennCNV and CNAM but was not validated in all calls made only by one program, was considered to have passed validation if the validated samples yielded an odds ratio greater than 2.0 with at least two cases confirmed by validation.

**Table 3:** Validated CNVs discovered using affected children from Utah families.

TaqMan validated Utah and sequence SNP CNV regions of significance											
CNV Origin	Cytoband	CNV Region - Discovery Cohort	CNV Region - Replication Cohort	CNV Type	Total Cases	Total Controls	OddsRatio	P Value	Cases	Controls	Gene/Region
Utah CNV	1q21.1	chr1:145714421-146101228	chr1:145703115-145736438	Dup	1542	5754	3.37	9.60E-03	9	10	CD160, PDZK1
Utah CNV	1q41	chr1:215858193-215861879	chr1:215854466-215861792	Del	1540	5754	2.12	5.02E-03	22	39	USH2A
Utah CNV	2p16.3	chr2:51272055-51336043	chr2:51266798-51339236	Del	1542	5755	14.96	8.26E-03	4	1	upstream of NRXN1
Utah CNV#	3q26.31	chr3:172596081-172617355	chr3:172591359-172604675	Dup	1540	5754	3.74	2.11E-01	1	1	downstream of SPATA16
Utah CNV#	4q35.2	chr4:189084983-189117429	chr4:189084240-189117031	Del	1544	5762	3.74	1.98E-01	2	2	downstream of TRIML1
Utah CNV#	6p24.3	chr6:7425246-7464367	chr6:7461346-7470321	Del	1544	5762	∞	2.11E-01	1	0	between RIOK1 and DSP
Utah CNV#	6q11.1	chr6:62443739-62462295	chr6:62426827-62472074	Dup	1544	5762	3.74	1.98E-01	2	2	KHDRBS2
Utah CNV	6q24.3	chr6:147588752-147664671	chr6:147577803-147684318	Del	1533	5751	∞	2.10E-01	1	0	STXBP5
Utah CNV#	7p22.1	chr7:6838712-6864071	chr7:6870635-6871412	Dup	1544	5762	7.47	1.15E-01	2	1	upstream of CCZ1B
Sequence SNP CNV#	7q21.3	Not found	chr7:93070811-93116320	Del	1544	5762	∞	4.46E-02	2	0	CALCR, MIR653, MIR489
Utah CNV#	9p21.1	chr9:28190069-28347679	chr9:28207468-28348133	Del	1544	5761	3.74	6.72E-02	4	4	LINGO2
Utah CNV#	9p21.1	chr9:28190069-28347679	chr9:28354180-28354967	Del	1544	5762	3.73	3.78E-01	1	1	LINGO2 (intron)
Utah CNV	10q23.1	chr10:83893626-84175018	chr10:83886963-83888343	Del	1505	5640	3.76	1.54E-02	7	7	NRG3 (intron)
Utah CNV#	10q23.31	chr10:92274764-92289762	chr10:92262627-92298079	Dup	1544	5761	7.47	1.15E-01	2	1	downstream of BC037970
Utah CNV#	12q23.2	chr12:102097012-102106306	chr12:102095178-102108946	Dup	1544	5762	7.47	1.15E-01	2	1	CHPT1
Utah CNV#	13q13.3	chr13:40087689-40088007	chr13:40089105-40090197	Del	1544	5761	∞	2.11E-01	1	0	LHFP (intron)
Sequence SNP CNV#	14q32.2	Not found	chr14:100705631-100828134	Dup	1544	5762	9.36	5.99E-03	5	2	SLC25A29, YY1, MIR345, SLC25A47, WARS
Sequence SNP CNV#	14q32.31	Not found	chr14:102018946-102026138	Dup	1544	5762	4.62	1.01E-14	60	50	DIO3AS, DIO3OS
Sequence SNP CNV#	14q32.31	Not found	chr14:102729881-102749930	Del	1544	5762	7.47	1.15E-01	2	1	MOK
Sequence SNP CNV#	14q32.31	Not found	chr14:102973910-102975572	Dup	1544	5762	3.82	8.29E-26	136	142	ANKRD9 (RAGE)
Sequence SNP CNV	15q11.2-q13.1	Not found	chr15:25690465-28513763	Dup*	1544	5762	41.05	1.82E-08	11	1	ATP10A, GABRB3, GABRA5, GABRG3, HERC2
Sequence SNP CNV#	15q13.2–15q13.3	Not found	chr15:31092983-31369123	Del	1543	5761	∞	4.46E-02	2	0	FAN1, MTMR10, MIR211, TRPM1
Sequence SNP CNV#	15q13.3	Not found	chr15:31776648-31822910	Dup	1544	5762	4.40	6.91E-06	21	18	OTUD7A
Sequence SNP CNV#	20q11.22	Not found	chr20:32210931-32441302	Dup	1544	5762	2.72	3.16E-02	8	11	NECAB3, CBFA2T2, C20orf144, NECAB3, C20orf134, PXMP4, NECAB3, ZNF341, E2F1, CHMP4B

CNVs shown here were selected based on their p value, their case/control odds ratio, or both and were subject to molecular validation.

* This CNV is contiguous with the chromosome 15q11.2 CNV described in Table 4 based on TaqMan data.

# Designates CNVs not previously seen in ASD, based on queries for genes included in or flanking the CNV.

**Figure 3:**
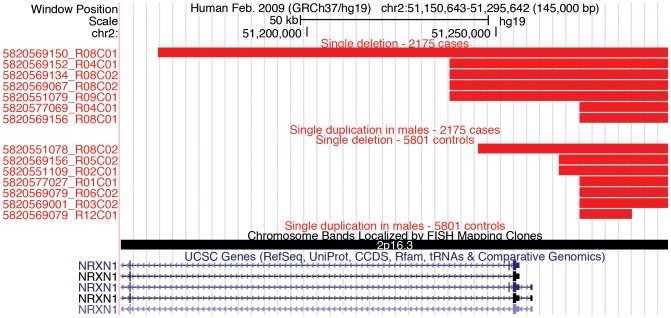
UCSC Genome browser view of CNVs in the *NRXN1* region. CNVs observed in the vicinity of the *NRXN1-alpha* transcription start site are shown. Note that most CNVs observed in ASD patients include exon 1 of *NRXN1-alpha* while only 1 control CNV extends into exon 1. Produced with custom tracks listing CNV calls and uploaded to http://genome.ucsc.edu.

Notable among these CNVs is a deletion observed near the 5′-end of the *NRXN1* gene. This deletion, observed in five cases and only in one control, includes at least a portion of the *NRXN1*-alpha promoter, and extends into the first exon of *NLRXN1-*α, as shown in the UCSC Genome Browser view [35] (Figure 3). CNVs impacting *NRXN1* in ASD as well as other neurological conditions have been published by others [15], [32], [36]–[40], so the observation of *NRXN1* CNVs both in our high-risk ASD family discovery work and in the large case/control replication study demonstrates our ability to detect biologically relevant CNVs that may also have clinical utility.

Other CNVs of interest included portions of the *LINGO2* and *STXBP5* genes. Single nucleotide variants in the *LINGO2* gene have been associated with essential tremor and with Parkinson's disease, suggesting that the LINGO2 protein may have a neurological function [41]. However, CNVs in this gene have not previously been identified in individuals with ASD. We also observed deletions involving a portion of the *STXBP5* gene, an interesting finding based on the potential role of STXBP5 in neurotransmitter release [42], [43].

### CNVs Identified by SNV Probes

Twenty-five additional CNVs shown in Table 3 were discovered using SNVs identified in our high-risk ASD families. The SNVs that detected these twenty-five CNVs (Table S4, File S1) were identified by exon capture and DNA sequencing in regions of haplotype sharing and in published ASD candidate genes in our high-risk ASD families, and were selected for further study because they might alter the function of the proteins in which they were found (unpublished observations). The 9 validated CNVs derived from SNV intensity data are shown in Table 3 (CNVs not detected in discovery cohort). One of these CNVs, a chromosome 15q duplication, encompasses three duplication CNVs in Table 4. These three CNVs are thought to be contiguous since TaqMan data confirmed the same samples to be positive for each of them.

**Table 4:** Published CNVs observed in our sample population.

Cytoband	Literature CNVs	Region of Highest Significance	CNV Type	TaqMan Validation	Total Cases	Total Controls	OddsRatio	P Value	Cases	Controls	Gene/Region
1q21.1	chr1:146555186-147779086	chr1:146656292-146707824	Dup	NT	1543	5761	7.48	1.15E-01	2	1	FMO5
2p24.3	chr2:13202218-13248445	chr2:13203874-13209245	Del	Validated (chr2:13203874-13209245)	1544	5761	∞	2.11E-01	1	0	upstream of LOC100506474
2p21	chr2:45455651-45984915	chr2:45489954-45492582	Dup	NT	1541	5756	∞	4.46E-02	2	0	between UNQ6975 and SRBD1
2p16.3	chr2:50145644-51259671	chr2:51237767-51245359	Del	NT	1544	5762	∞	1.99E-03	4	0	NRXN1
2p15	chr2:62258231-63028717	chr2:62230970-62367720	Dup	NT	1543	5762	∞	2.11E-01	1	0	COMMD1
2q14.1	chr2:115139568-115617934	chr2:115133493-115140263	Del	NT	1543	5759	7.47	1.15E-01	2	1	between LOC440900 and DPP10
3p26.3	chr3:1940192-1940920	chr3:1937796-1941004	Del	Validated (chr3:1937796-1942764)	1544	5760	5.60	6.70E-02	3	2	between CNTN6 and CNTN4
3p14.1	chr3:67656832-68957204	chr3:67657429-68962928	Del	NT	1544	5762	∞	2.11E-01	1	0	SUCLG2, FAM19A4, FAM19A1
4q13.3	chr4:73756500-73905356	chr4:73766964-73816870	Dup	Validated (chr4:73753294-74058988)	1544	5760	∞	2.11E-01	1	0	COX18, ANKRD17
4q33	chr4:154087652-172339893	chr4:171366005-171471530	Del	NT	1543	5761	∞	4.46E-02	2	0	between AADAT and HSP90AA6P
5q23.1	chr5:118478541-118584821	chr5:118527524-118589485	Dup	Validated (chr5:118527524-118614781)	1541	5760	3.74	1.98E-01	2	2	DMXL1, TNFAIP8
6p21.2	chr6:39071841-39082863	chr6:39069291-39072241	Del	Validated (chr6:39069291-39072241)	1544	5759	2.37	1.93E-02	12	19	SAYSD1
8q11.23	chr8:54858496-54907579	chr8:54855680-54912001	Dup	Validated (chr8:54855680-54912001)	1544	5762	∞	2.11E-01	1	0	RGS20, TCEA1
10q11.22	chr10:46269076-50892143	chr10:49370090-49471091	Dup	NT	1528	5750	3.77	1.96E-01	2	2	FRMPD2P1, FRMPD2
10q11.23	chr10:50892146-51450787	chr10:50884949-50943185	Dup	NT	1542	5760	3.74	1.98E-01	2	2	OGDHL, C10orf53
12q13.13	chr12:53183470-53189890	chr12:53177144-53180552	Del	Validated (chr12:53177144-53182177)	1544	5762	∞	4.46E-02	2	0	between KRT76 and KRT3
15q11.1	chr15:20266959-25480660	chr15:20192970-20197164	Dup	Validated (chr15:20192970-20212798)	1515	5632	4.97	4.06E-02	4	3	downstream of HERC2P3
15q11.2	chr15:20266959-25480660	chr15:25099351-25102073	Del	NT	1540	5761	3.75	1.13E-01	3	3	SNRPN
15q11.2	chr15:20266959-25480660	chr15:25099351-25102073	Dup	NT	1541	5759	45.19	7.93E-08	12	1	SNRPN
15q11.2	chr15:25582397-25684125	chr15:25579767-25581658	Dup*	Validated (chr15:25576642-25581880)	1540	5761	∞	3.86E-06	8	0	between SNORD109A and UBE3A
15q11.2	chr15:25582397-25684125	chr15:25582882-25662988	Dup*	NT	1540	5762	30.08	2.82E-05	8	1	UBE3A
16p12.2	chr16:21901310-22703860	chr16:21958486-22172866	Dup	NT	1544	5761	∞	4.47E-02	2	0	C16orf52, UQCRC2, PDZD9, VWA3A
16p11.2	chr16:29671216-30173786	chr16:29664753-30177298	Del	NT	1544	5761	7.47	1.15E-01	2	1	DOC2A, ASPHD1, LOC440356, TBX6, LOC100271831, PRRT2, CDIPT, QPRT, YPEL3, PPP4C, MAPK3, SPN, MVP, FAM57B, ZG16, ALDOA, INO80E, SEZ6L2, TAOK2, KCTD13, MAZ, KIF22, GDPD3, C16orf92, C16orf53, TMEM219, C16orf54, HIRIP3
16q23.3	chr16:82195236-82722082	chr16:82423855-82445055	Dup	NT	1542	5758	∞	4.46E-02	2	0	between MPHOSPH6 and CDH13
17p12	chr17:14139846-15282723	chr17:14132271-14133349	Dup	Validated (chr17:14132271-14133568)	1544	5762	1.60	3.57E-01	3	7	between COX10 and CDRT15
17p12	chr17:14139846-15282723	chr17:14132271-15282708	Del	NT	1544	5761	5.61	6.70E-02	3	2	PMP22, CDRT15, TEKT3, MGC12916, CDRT7, HS3ST3B1
17p12	chr17:14139846-15282723	chr17:14952999-15053648	Dup	NT	1543	5760	3.74	1.98E-01	2	2	between CDRT7 and PMP22
17p12	chr17:14139846-15282723	chr17:15283960-15287134	Del	Validated (chr17:15283960-15287134)	1544	5761	3.74	1.13E-01	3	3	between TEKT3 and FAM18B2-CDRT4
20p12.3	chr20:8044044-8527513	chr20:8162278-8313229	Dup	NT	1544	5761	3.73	1.98E-01	2	2	PLCB1
Xp21.2	chrX:28605682-29974014	chrX:29944502-29987870	Dup	NT	1544	5760	∞	4.47E-02	2	0	IL1RAPL1
Xq27.2	chrX:139998330-140443613	chrX:140329633-140348506	Del	Validated (chrX:140329633-140456325)	1544	5762	7.48	2.06E-02	4	2	SPANXC
Xq28	chrX:148858522-149097275	chrX:148882559-148886166	Del	Validated (chrX:148882559-149020410)	1540	5754	∞	4.46E-02	2	0	MAGEA8

* Denotes CNVs contiguous with the chromosome 15q11.2–13.1 CNV shown in Table 3.

**Figure 4:**
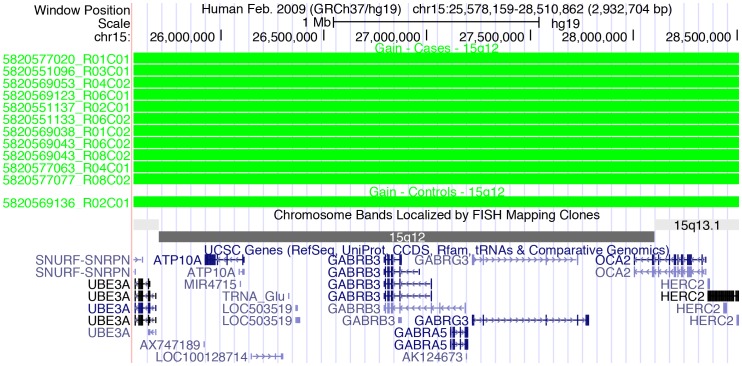
UCSC Genome Browser View of CNVs in the GABR Region on chromosome 15q12. Duplications were called by both PennCNV and by CNAM in this region, however the number of duplications called by each program differed, with many additional duplications called by CNAM. Produced with custom tracks listing CNV calls and uploaded to http://genome.ucsc.edu.

Interestingly, duplications involving the GABA receptor gene cluster, as well as many other genes, on chromosome 15q12 were observed in 11 unrelated cases in our study and only in a single control, shown in the UCSC Genome Browser view [35](Figure 4). Contrary to our findings, a recent search for CNVs in GABA pathway genes [44] did not find an enrichment of duplications in this region. Rather, both deletions and duplications were observed at similar frequencies in cases and controls.

### Published CNVs

Additional CNVs from the literature and both published and unpublished CNVs identified at CHOP also were observed in our large dataset and met our criteria for potential clinical utility. Of those, 31 high-impact CNVs are shown in Table 4. All CNVs not previously experimentally validated were validated in this study.

One of the previously unpublished CHOP CNVs is a duplication that encompasses the 3′-end *RGS20* gene as well as the 3′-end of the *TCEA1* gene. The RGS gene family encodes proteins that regulate G-protein signaling. These proteins function by increasing the inherent GTPase activity of their target G-proteins, and thus limit the signaling activity of their target G-proteins by keeping them in the inactive, GDP-bound state. *RGS20* is expressed throughout the brain (reviewed in [45]), making it a likely candidate for involvement in neurological development. The *TCEA1* gene, which also is partially encompassed by this CNV, is a transcription elongation factor involved in RNA polymerase II transcription. A role for TCEA1 in cell growth regulation has been suggested [46]. This potential role is consistent with the involvement of *TCEA1* CNVs in ASD etiology as well.

### Pathway analysis

Analysis of 104 genes within or immediately flanking our PCR-validated CNVs yielded significant association of these genes to previously characterized functional networks. The five most statistically significant networks, along with their statistical scores, are shown in Table 5. The top ranking functional categories identified in this analysis, along with their P-values, are shown in Table 6.

**Table 5:** Top Significant Networks Identified by Pathway Analysis using Ingenuity IPA.

Network	Score
Cell-To-Cell Signaling and Interaction, Tissue Development, Gene Expression	55
Neurological Disease, Behavior, Cardiovascular Disease	28
Cell Death, Cellular Compromise, Neurological Disease	26
Cellular Development, Cell Morphology, Nervous System Development and Function	20
Behavior, Cardiovascular Disease, Neurological Disease	18

Network scores are the –log P for the results of a right-tailed Fisher's Exact Test.

**Table 6:** Top Significant Biological Functions identified by Ingenuity IPA and literature searches.

Function	p-value range	# Genes
Neurological Disease	2.71E-05 - 3.15E-02	14 (18)
Behavior	5.93E-05 - 4.36E-02	10
Cardiovascular Disease	8.58E-05 - 4.30E-02	10
Cellular Development	1.39E-04 - 4.77E-02	9
Inflammatory response	4.84E-04 - 2.89E-02	6

The right-tailed Fisher's exact test was used to calculate P-values representing the probability that selecting genes associated with that pathway or network is due to chance alone. Each functional category represents a collection of associated subcategories, each of which has an associated P-value. For example, within ‘Neurological Disease,’ are subcategories of genes associated with seizures, Huntington Disease, schizophrenia, etc. The P-value range given represents the range of P-values generated for each subcategory. In the first line, 14 genes were associated with a function in Neurological Disease by Ingenuity software. An additional 4 genes were identified as having neurological functions in the literature, giving a total of 18 with known or suspected roles in neurological disease.

As expected for CNVs associated with a neurodevelopmental disorder, a significant number of genes in or adjacent to the CNVs described here are involved in neural function, development and disease (Tables 5–6). Examples of such genes include: *GABRA5*, *GABRA3*, *GABRG3*, *UBE3A*, *E2F1*, *PLCB1*, *PMP22*, *AADAT*, *MAPK3*, *NRXN1*, *NRG3*, *DPP10*, *UQCRC2*, *USH2A*, *NECAB3*, *CNTN4*, *LINGO2*, *IL1RAPL1*, *STXBP5*, DOC2A, and *SNRPN*. Of these genes, *E2F1*, *AADAT*, *NECAB3*, and *IL1RAPL1* are not found in the Autism Chromosome Rearrangement Database (http://projects.tcag.ca/autism/), suggesting that they may be novel ASD risk genes.

The novel ASD risk loci identified here have functions that suggest a significant role in brain function and architecture. As such, altering the function of each of these genes as a result of the CNV could impinge on the biochemical pathways that are relevant to ASD etiology.

For example, mutations in *IL1RAPL1* have been observed in cases of X-linked intellectual disability [47], and the encoded protein has been shown to play a role in voltage-gated calcium channel regulation in cultured cells [48]. *E2F1* encodes a transcription factor and DNA-binding protein that plays a significant role in regulating cell growth and differentiation, apoptosis and response to DNA damage (reviewed in Biswas and Johnson, 2012 [49]). Each of these genes thus could have detrimental impacts on normal brain function.

*NECAB3* encodes a neuronal protein with two isoforms that regulate the production of beta-amyloid peptide in opposite directions, depending on whether exon 9 of *NECAB3* is included in or excluded from the mature mRNA [50].

*AADAT* encodes an aminotransferase with multiple functions, one of which leads to the synthesis of kynurenic acid. This pathway has been proposed as a target for potential neuroprotective therapeutics, indicating the potential significance of this finding for ASD etiology (reviewed in Stone et al., 2012 [51]). The specific roles that any of these genes play in ASD etiology have yet to be determined, but the observed neurological functions of their encoded proteins strongly support a potential role in normal brain function.

Many of these genes also have been implicated in other nervous system disorders, including Huntington's, Parkinson's, and Alzheimer's diseases as well as schizophrenia and epilepsy [41], [52]–[61]. One of the features common to this group of disorders, which includes ASD, is synaptic dysfunction. There is a significant overlap in genes, and/or the molecular mechanisms by which these genes give rise to synaptopathies (reviewed in [62]). We therefore find it notable that many such genes involved in other synaptopathies were found within or flanking the validated CNVs we identified as associated with ASD.

In addition to neurogenic genes, validated CNVs were associated with genes with known roles in renal and cardiovascular diseases (Table 6). Several syndromic forms of autism, such as DiGeorge Syndrome and Charcot-Marie Tooth Disease are comorbid with renal and cardiovascular disease, and therefore it was not surprising to find that our study identified CNVs containing genes associated with these syndromes and functions, such as *CDRT15*, and *CDH13*.

There is mounting evidence, as well, that inflammatory responses are involved with the development and progression of autism (reviewed in [63]). Maternal immune activation during pregnancy is believed to activate fetal inflammatory responses, in some cases with detrimental effects on neural development in the fetus, leading to autism. This environmental insult could be mediated or enhanced by genomic changes that predispose the fetus to elevated inflammatory responses, so it is significant that a number of genes from our validated CNVs play a role in inflammatory response. Examples of these include *CD160*, *CALCR*, and *SPN*.

Our findings are consistent with other studies that used pathway analysis to characterize the genes contained in ASD risk CNVs, and suggest that many different biological pathways, when disrupted, can lead to features observed in ASD. The wide variety of biological functions identified for these genes also is consistent with estimates of the number of independent genetic variants that may play a role in the etiology of ASD (8–11).

## Discussion

We used a custom microarray to characterize the frequency of CNVs identified in high-risk ASD families in a large ASD case/control population. We also evaluated further the frequency of CNVs discovered in several published studies in our sample cohort to obtain a clearer picture of the potential clinical utility of these CNVs in the genetic evaluation of children with ASD. We used multiple quality control measures to insure that all cases and controls a) had no unexpected familial relationships; b) represented a uniform ethnic group; c) were devoid of uncharacterized whole chromosome anomalies or other genomic abnormalities consistent with syndromic forms of ASD; d) had sufficient power to distinguish risk variants from CNVs with little or no impact on the ASD phenotype; and e) were validated using quantitative PCR even though the custom array used here represented at least a second evaluation for most of them. Parents of ASD cases tested were not available to determine state of inheritance.

The validity of our approach was confirmed by our observation of CNVs that had been previously identified as ASD risked markers, including CNVs encompassing parts of the *NRXN1* gene. CNVs and point mutations in *NRXN1* are thought to play a role in a subset of ASD cases as well as in other neuropsychiatric conditions [15], [32], [36]–[40]. The data from our study demonstrate that *NRXN1* CNVs also occur in high-risk ASD families. Further, our case/control data provide additional evidence that neurexin-1 plays an important role in unrelated ASD cases. While CNVs near *NRXN1* occur in controls as well as in cases, the CVNs observed in our ASD cases typically disrupt a portion of the *NRXN1* coding region while CNVs observed in our control population do not.

### CNVs from high-risk ASD families

In our high-risk ASD families, we identified both novel and previously observed CNVs containing genes with potential relevance to neuropsychiatric conditions such as ASD. These include CNVs involving *LINGO2*, the GABR gene cluster on chromosome 15q12 and *STXBP5*. Each of these CNV regions has an odds ratio greater than 2 and most of the CNVs we identified in high-risk families have a significant p value associating them with the ASD phenotype in this case/control study. Some CNVs, although observed only in ASD cases and not in controls, were too rare even in this large dataset to generate statistically significant results. An example is a deletion involving *STXBP5* that was observed two ASD samples and in no controls. A deletion including this gene was previously observed in a patient with an apparent syndromic form of ASD [64], lending further support to our observation of *STXBP5* deletions in ASD cases. These data collectively suggest that CNVs observed in high-risk ASD families also are important contributors to the etiology of ASD in an ASD case/control population.

We detected rare duplications involving the GABA receptor gene cluster as well as additional genes in the Prader-Willi/Angelman syndrome region on chromosome 15 (11/1,544 unrelated cases, 1/5,762 unrelated controls, OR = 40.05). All of these CNVs were confirmed using TaqMan assays spanning the region, and these results strongly suggest a role for duplications on chromosome 15q12 in ASD etiology. Deficiency of GABA_A_ receptors indeed is thought to play an important role in both autism and epilepsy, and duplications have been observed to result in decreased GABR expression through a potential epigenetic mechanism (reviewed in [65]). Further, differences in the expression of *GABRB3* mRNA and protein in the brains of some children with autism have been reported along with loss of biallelic expression of the chromosome 15q GABR genes in some individuals, [66], suggesting that epigenetic regulation of the chromosome 15 GABR gene cluster could also contribute to ASD etiology. Consistent with many previous findings from family studies, case reports and modest case/control studies (http://omim.org/entry/608636), our data provide additional support for the involvement of duplications in this region of the genome in ASD. Further, our large population study suggests that these duplications may explain as much as 0.7% of ASD cases.

A recent study searching for CNVs encompassing genes in the GABA pathway, including the chromosome 15 GABR gene cluster, also found CNVs in this region. In contrast to our findings, this study found GABR gene cluster duplications at similar frequencies in both cases and in controls (Table S2 in ref. [44]). In addition, deletions were more common in this study in both cases and controls, while duplications were more common in our data. The differences between the two studies may lie in the sample population being studied, the uniformity of our sample population, or the technology platform used for CNV discovery (custom Illumina array compared to a custom Agilent array). Previous results have demonstrated maternal inheritance of deletions in this region in children with autism [67]. However, in our family studies we did not observe CNVs involving chromosome 15q12, and our case/control data preclude us from determining the parent of origin.

Interestingly, the CNVs that we observed on chromosome 15q were detected primarily with probes for SNVs identified in the GABR genes. Further, these SNVs were identified in affected individuals from high-risk ASD families. We did not observe CNVs involving this region in our high-risk ASD families. The observation of frequent duplications in our case/control population in the region containing these genes, coupled with the detection of these CNVs using probes for potential detrimental single nucleotide variants, suggests that both SNVs and CNVs involving the GABR genes might be pathogenic.

### Literature supported CNVs

In addition to the CVNs identified in our high-risk ASD families, we evaluated further ASD risk CNVs identified in previous studies. Our results (Table 4) clearly demonstrate a role for many of these CNVs in ASD pathogenesis. Consistent with previous results, our data demonstrate in a large ASD population that rare CNVs are likely to play a role in the genetics of ASD, and suggest that these CNVs should be included in the genetic evaluation of children with ASD.

Interestingly, recent publications have identified a recurrent duplication of the Williams syndrome region on chromosome 7q11.23 in children with ASD [9], [11]. We included probes for this region on our custom array, and were not able to identify any 7q11.23 duplications in our datasets. The reason(s) we did not observe any duplications in this region is not obvious; we had adequate probe coverage to have seen such duplications if they were present. Similar to the simplex ASD families used in those published studies, most of our ASD samples also were from reported simplex families, so the lack of observation of these CNVs is unlikely to be due to differences in family structure.

A CNV discovered at CHOP and not previously published includes a portion of the LCE gene cluster on chromosome 1. Deletions in this region have been associated with psoriasis [68], [69], but no variants in this region have been linked to autism. Focusing solely on individuals of northern and western European ancestry, we observed this CNV deletion in a single case and also a single control. However, when we included samples of non- European or uncertain ancestry, we observed 27 additional case DNA samples that carried this deletion, while only a single additional CNV-positive control was observed. Interestingly, based on SNP genotype results from principal component analysis, all of the cases that were positive for this CNV were of Asian descent. Since our control cohort had few individuals of Asian descent, we suspected that this CNV might be common in the Asian population. Analysis of whole genome data for individuals of non- European ancestry genotyped at the Center for Applied Genomics did not demonstrate common CNVs in either cases or controls in this region in individuals with Asian ancestry. However, a common CNV including LCE3E was observed in individuals with African ancestry (unpublished observations). Further analysis will be necessary to determine if this CNV is an ASD risk variant in either Asian or African populations.

### Effect of analysis method on CNV validation

Although some CNVs are described here for the first time, many of the CNVs that we evaluated in this study were described previously. It is interesting to note that individual CNV calls that were made with both of the software packages we used were much more likely to be validated by qPCR than were CNVs called by either program alone. In fact, 97% of the CNVs called by both PennCNV and CNAM validated using TaqMan qPCR assays, while only 24% of the CNVs called by PennCNV alone and 30% of the CNVs called by CNAM alone were validated using the same approach. The concordance between the two analysis methods is informative given that the final sample sets used by the two methods differed substantially. The CNAM analysis used 290 fewer case samples and 575 fewer control samples than the PennCNV analysis. These data clearly demonstrate the value of using multiple software packages to evaluate microarray data for CNV discovery work. Our data are consistent with the rarity of many CNVs detected in DNA from children with ASD, and with the suggestion that there may be hundreds of loci that contribute to the development of ASD [9], [11].

Our data demonstrate that CNVs identified in high-risk ASD families play a role in the etiology of ASD in unrelated cases. Evaluation of these CNVs in the large sample set used in this study provides compelling evidence for extremely rare recurrent CNVs as well as additional common variants in the genetics of ASD. We suggest that the CNVs described here likely have a strong impact on the development of ASD. Given the extensive quality control measures we used to characterize our sample cohort, the frequency at which we observed these CNVs in our cohort, and the molecular validation that we used to verify the calls, these CNVs can be used to increase sensitivity in the genetic evaluation of children with ASD. Further work will help to determine if the CNVs reported here are important for specific clinical subsets of ASD cases.

## Materials and Methods

### Ethics statement

The research presented here has been approved by the University of Utah Institutional Review Board (IRB) (University of Utah IRB#:6042-96) and the Children's Hospital of Philadelphia IRB (CHOP IRB#: IRB 06-004886). Patients and their families were recruited through the University of Utah Department of Psychiatry or the Children's Hospital of Philadelphia clinic or CHOP outreach clinics. Written informed consent was obtained from the participants or their parents using IRB approved consent forms prior to enrollment in the project. There was no discrimination against individuals or families who chose not to participate in the study. All data were analyzed anonymously and all clinical investigations were conducted according to the principles expressed in the Declaration of Helsinki.

### DNA samples

DNA samples from high-risk ASD family members were collected through the University of Utah Department of Psychiatry. Three independent sample cohorts, comprising 3,000 ASD patient samples (72% male), were collected for CNV replication. Of those, 857 samples were from probands recruited and genotyped by the Center for Applied Genomics (CAG) at CHOP from the greater Philadelphia area using a CHOP IRB-approved protocol; 2,143 ASD samples were from the AGRE and the AGP consortium (Rutgers, NJ ASD repository), and genotyped at the CAG center at CHOP (Table 1). Only samples from affected individuals diagnosed using the Autism Diagnostic Interview-Revised (ADI-R) and the Autism Diagnostic Observation Schedule (ADOS) were used in the study. All control samples were from CHOP and were matched in a 2∶1 ratio with the ASD cases.

### CNV Discovery in high-risk ASD families

DNA samples were genotyped on the Affymetrix Genome-Wide Human SNP Array 6.0 according to the manufacturer's protocol. Fifty-five autism subjects were chosen from 9 families with multiple affected first-degree relatives. The number of individuals with an autism diagnosis in these families ranged from 3 to 9. Affected individuals were diagnosed using ADI-R and ADOS. Control subjects (N = 439) for the discovery phase of the project were selected from Utah CEPH/Genetics Reference Project (UGRP) families [70]. All microarray experiments were performed on blood DNA samples, except for two of the 55 case samples and three control subjects for which DNA from lymphoblastoid cell lines was used. CNVs were initially detected using the Copy Number Analysis Module (CNAM) of Golden Helix SNP & Variation Suite (SVS) (Golden Helix Inc.). Log ratios were calculated by quantile normalizing the A allele and B allele intensities using the entire population as a reference median for each SNP.

Batch effects in the log ratios were corrected via numeric principle component analysis (PCA) [71]. CNV segmentation analysis was carried out for each individual using the univariate CNAM segmentation procedure of Golden Helix SVS. We used a moving window of 5,000 markers, maximum number of segments per window of 20, minimum segment size 10 markers, and pairwise permutation p-value of 0.001.

### iSelect array design

Probes for each CNV to be characterized in this study were selected from the Illumina Omni2.5 array probe set. Probes were selected to be as uniformly spaced across each region and flanking each region as possible (using the hg19 genome build). For each CNV, we included 10 or more probes within the defined CNV region (CNVr) and five probes on each flank (except where not possible due to the telomeric location of a CNVr). Probes for an additional 185 CNVs described in the literature, including 104 identified by CHOP in samples that partially overlap those used in this study, also were included for further CNV validation. We attempted to increase probe coverage for CNVs identified with only a small number of probes. Finally, we included probes for 2,799 putative functional candidate SNVs detected by targeted exome DNA sequencing on 26 representative individuals from 11 ASD families (unpublished data). The genes that we targeted for exome sequencing included all known genes in regions of familial haplotype sharing and linkage as well as additional autism candidate genes. These SNVs, although included in a search for potential ASD point mutations, also were used to identify additional CNVs.

### Array processing

We performed high throughput SNP genotyping using the Illumina Infinium™ II BeadChip technology (Illumina, San Diego), at the Center for Applied Genomics at CHOP. Detailed methods for array processing are available in the associated Supplemental Materials.

### CNV calling and statistical analysis

CNVs were called using both PennCNV [34] [35] and CNAM (Golden Helix SNP & Variation Suite (SVS), Golden Helix, Inc.). CNV calling using PennCNV was performed as described [32]. For CNAM calls, we chose not to examine whole chromosomes, but rather to analyze each target region separately. Since our array targeted specific regions and did not have probe coverage over much of the genome, it was desirable to avoid calling segments that spanned large regions with no data, and prevent any CNV calls from being influenced by distant data points. To accomplish this, the markers in the data set were grouped into “pseudochromosomes”, one for each CNVr covered by the array, that were then considered individually in the segmentation algorithm. CNAM was run using the univariate option with no moving window, maximum of 5 segments per pseudochromosome, minimum segment size of 1 marker, and permutation p-value threshold of 0.001. After segmentation, we classified segments as losses, gains, or neutral. Fisher's exact test was used to test for association of copy number loss vs. no loss, and copy number gain vs. no gain. Similar tests were conducted for the X chromosome, stratified by gender. Odds ratios also were calculated as an indicator of potential clinical risk for each CNV.

### Laboratory confirmation of CNVs

Array results were confirmed using pre-designed Applied Biosystems TaqMan copy number assays or custom-designed TaqMan copy number assays when necessary (Life Technologies, Inc.). All CNVs with odds ratios greater than 2.0 and present in at least two cases were selected for molecular validation. We did not select CNVs with odds ratios less than 2 for validation since we wanted to validate only those with high potential clinical utility. Six CNVs also were selected for validation because they were adjacent to, but not overlapping, literature CNVs that were covered by probes on the custom array. A maximum of 6 case samples were validated for each CNV. Five negative control samples, selected based on their lack of all of the CNVs under study also were included in each validation assay. A list of all of the TaqMan assays used in this work is found in Table S1 in File S1, and detailed procedures are described in File S2.

### Pathway analysis

Analysis of biological pathways encompassing genes found in the CNV regions was performed using the bioinformatics tools DAVID Bioinformatics Resources 6.7 [72], [73] and Ingenuity Pathways Analysis (IPA) (Ingenuity® Systems). We performed network and pathway analyses on genes contained within the CNVs or immediately flanking intergenic CNVs that were PCR validated. Pathway analysis details are described in File S2.

## Supporting Information

File S1 **Supplemental data Tables S1–S4.** **Table S1.** **TaqMan assays used for CNV confirmation.** Genomic coordinates and assay names for all TaqMan assays used for CNV confirmation. All coordinates are shown using hg19 coordinates. **Table S2. CNVs identified in Utah ASD families.** The chromosomal locations and copy number status of all CNVs identified in Utah ASD families are shown. If CNVs had been observed in previous studies, the references are given below the table. **Table S3. Literature CNVs.** Chromosomal coordinates (hg19), source, and probe coverage on the custom Illumina array for each literature CNV characterized in this study. **Table S4.** **CNVs identified using probes for sequence variants identified in Utah ASD families.** Chromosome coordinates, TaqMan validation status, and copy number status for all CNVs identified using probes to sequence variants identified in Utah ASD families are shown. For one CNV the single TaqMan assay that was used failed PCR quality control.
(XLSX)

File S2 **Supplemental methods and results. Supplemental methods. Details regarding sources of DNA samples used, methods for array processing, and sample quality control are described. Supplemental results. Analysis of population stratification using principal component analysis.** The only samples used in CNV analyses were those demonstrated to be within the Caucasian group. His analysis included all cases and controls genotyped in this study.
(DOCX)
