# Supplementary material for: Identification of Rare Recurrent Copy Number Variants in High-Risk Autism Families and Their Prevalence in a Large ASD Population
Source: PLoS One. 2013 Jan 14;8(1):e52239. doi: 10.1371/journal.pone.0052239 (PMC3544904; doi:10.1371/journal.pone.0052239)
Supplement: File S2 — Supplemental methods and results. Supplemental methods. Details regarding sources of DNA samples used, methods for array processing, and sample quality control are described. Supplemental results. Analysis of population stratification using principal component analysis. The only samples used in CNV analyses were those demonstrated to be within the Caucasian group. His analysis included all cases and controls genotyped in this study. (DOCX) [file pone.0052239.s002.docx]

**Identification of Copy Number Variants in High-Risk Autism Families and their Prevalence in a Large ASD Population**

**Nori Matsunami**^1^, **Dexter Hadley**^2^**, Charles Hensel**^3^**, Bryce Christensen**^4^**, Jeff Stevens**^1^**, Lisa Baird**^1^**, Edward Frackelton**^2^**, Cecilia Kim**^2^**, Kelly Thomas**^2^**, Renata Pellegrino da Silva**^2^**, Brith Otterud^1^, Karen Ho**^3^**, Tena Varvil^1^, Tami Leppert^1^, Christophe G. Lambert**^4^**, Mark Leppert^1^, Hakon Hakonarson**^2,5^

**1** Department of Human Genetics, University of Utah, Salt Lake City, Utah, **2** Center for Applied Genomics, The Children’s Hospital of Philadelphia, Philadelphia, Pennsylvania, **3** Lineagen, Inc., Salt Lake City, Utah, **4** Golden Helix, Inc., Bozeman, Montana, 5 Department of Pediatrics, University of Pennsylvania School of Medicine, Philadelphia, Pennsylvania

**Supplemental Methods**

**Samples:** All high risk ASD family members and controls were of self-reported European ancestry. Among all cases in the replication study, 84% were of self-reported European ancestry, 6% were of self-reported African ancestry, 5% were self-reported as having multiple ethnic origins, and 5% were of unknown ethnicity. Among the cases, 1,577 were reported from unique families, 864 from 432 different families with 2 siblings, 369 from 123 different families with 3 siblings, 172 from 43 different families of 4 siblings, 5 siblings from a single family, 6 siblings from a single family, and 7 siblings from a single family. Among the DNA from cases used for genotyping, 1% came from cell pellets, 61% come from lymphoblastoid cell lines, 35% came from whole blood, and for 3% the source of DNA remained unknown. DNA was extracted from cell lines or lymphocytes, and quantitated using UV spectrophotometry. Six thousand controls were recruited by CHOP after obtaining informed consent under an IRB approved protocol. All DNA samples from controls were extracted from whole blood. Only individuals with self-reported Caucasian ancestry were used for this study. Pairwise identity by descent (IBD) was used to confirm known family assignments for cases, and to identify cryptic relatedness arising out of multiple subject enrollments across/within cohorts for all samples. Related individuals were removed so that only one family member remained in the study.

**Array processing:** We used 250ng of genomic DNA to genotype each sample, according to the manufacturer’s guidelines. On day one, genomic DNA was amplified 1000-1500-fold. Day two, amplified DNA was fragmented ~300-600bp, then precipitated and resuspended, followed by hybridization on to a BeadChip. Single base extension (SBE) utilizes a single probe sequence ~50bp long designed to hybridize immediately adjacent to the SNP query site. Following targeted hybridization to the bead array, the arrayed SNP locus-specific primers (attached to beads) were extended with a single hapten-labeled dideoxynucleotide in the SBE reaction. The haptens were subsequently detected by a multi-layer immunohistochemical sandwich assay, as recently described (Pastinen et al., 2000, Genome Res. 10, 1031, Erdogan et al., 2001, Nuc. Acids Res. 29, E36). The Illumina iScan was used to scan each BeadChip at two wavelengths and an image file was created. As BeadChip images were collected, intensity values were determined for all instances of each bead type, and data files were created that summarized intensity values for each bead type. These files were loaded directly into Illumina’s genotype analysis software, BeadStudio. A bead pool manifest created from the LIMS database containing all the BeadChip data was loaded into BeadStudio along with the intensity data for the samples. BeadStudio used a normalization algorithm to minimize BeadChip to BeadChip variability. Once the normalization was complete, the clustering algorithm was run to evaluate cluster positions for each locus and assign individual genotypes. Each locus was given an overall score based on the quality of the clustering and each individual genotype call was given a GenCall score. GenCall scores provided a quality metric that ranges from 0 to 1 assigned to every genotype called. GenCall scores were then calculated using information from the clustering of the samples. The location of each genotype relative to its assigned cluster determined its GenCall score.

**Sample quality control:** Quality control measures were intended to identify the samples with the greatest probability of successful CNV identification and to remove the samples with features making CNV identification problematic. Most of the QC metrics employed were originally designed for applications involving high-density genome-wide data. For this study, it was deemed possible that an otherwise high-quality sample with a few large CNVs might fail some QC metrics due to the sparse nature of the data from the custom array employed. The QC process was therefore approached with caution, and inclusion criteria were determined by manual review of the data for each metric in order to identify the outlier values. Details of data QC are available in the supplementary methods.

*Derivative log ratio spread (DLRS):* Derivative Log Ratio Spread (DLRS) is a measurement of point-to-point consistency of LR data, and is a reflection of the signal-to-noise ratio. It is similar in nature to the standard deviation of LR values that is often used in CNV studies, but has the advantage of being robust against large CNVs, which may influence standard deviation. DLRS was calculated for each chromosome, and the median chromosome DLRS value was used as a quality test. The distribution of the median DLRS statistic can be seen below. The outlier threshold was set at 0.3. One hundred twenty-eight subjects fail at this threshold, including all of the 75 samples that failed the waviness factor QC metric (see below).

*Waviness factor*: The “waviness” of each sample in the study was measured using the method of Diskin, et al. [27] as employed within SVS. An absolute value of 0.2 was determined as the outlier threshold for this metric, and 75 subjects failed at this threshold.

*Chromosomal Abnormalities and Cell-Line Artifacts:* Fifty-one samples (12 cases and 39 controls) were determined to have a chromosome 21 trisomy, consistent with a diagnosis of Down syndrome. These subjects were later confirmed to have Down syndrome based on clinical data review, and were removed from all further analyses. Additionally, 10 samples were removed based on other abnormalities that appeared to affect entire chromosomes.

*Excessive CNVs*: During the course of our analysis, several subjects were noted, using heat map style plots, to have a high frequency of copy number variant regions, in particular copy number gains. To identify the problematic subjects, we estimated the proportion of autosomal CNV regions in the data for which each subject had any CNV gain or loss. After manual review of the distribution of this proportion, 17 subjects with CNV calls at more than 10% of the regions were dropped from further analysis.

*Principal component analysis (PCA).* Substantial stratification was observed in the LR intensity data. The first two components were stratified by gender, and additional stratification and clustering was observed in the higher components as well. It was therefore considered prudent to apply a PCA correction to the intensity data prior to analysis in order to reduce the probability of data artifacts influencing CNV calls. The principal components were calculated based on all 9,000 samples in the QC process and the results were skewed by the presence of low quality samples. The principle components were therefore recalculated for the 8,777 samples passing preliminary QC, including samples that passed the tests for waviness, DLRS, PCA outliers, chromosome 21 trisomies, and the initial genotyping lab QC. After calculating the first 50 principal components and examining the distribution of eigenvalues, the LR values were corrected for 20 principal components, which were determined to be sufficient to explain the majority of variability in the data. The corrected LR data was then used for segmentation and CNV identification.

**CNV calling:** The segmentation covariates were reduced to a non-redundant spreadsheet, with columns for each marker position where at least one subject had an intensity shift. The distribution of values for each of these columns then was analyzed to determine if multiple copy number states were present, and if so, to estimate the threshold values that defined the different classes. The threshold values were first estimated by a simple algorithm that identified the mode of the distribution, and assuming this to be the neutral copy number state, set upper and lower thresholds based on the variance of the distribution. These thresholds were then manually reviewed, and gross errors were corrected as necessary. After threshold values were confirmed for each of the non-redundant regions, each subject’s data for that region was classified accordingly as loss, gain, or neutral. These values were then used to populate a table of discrete copy number calls for use in association testing.

**TaqMan assays:** DNA samples and controls were transferred from stock tubes and diluted with molecular grade water to a final concentration of 5ng/ul into 0.75mL Thermo Scientific Matrix storage tubes. All pipetting steps were carried out using Beckman Coulter Biomek FXp automation (Beckman Coulter, Inc., Fullerton, CA, USA) unless otherwise stated.  For each assay, 14ul of each sample were plated into rows of a 96-well full-skirted plate.  The last well in each row was left blank as a non-template control.  Each quadrant of the 384-well reaction plates was stamped with 2ul of DNA from the 96-well sample plate, so that each sample was assayed in quadruplicate.  The reaction plates were dried and stored at 4˚C.  The TaqMan® reaction mix for each assay was prepared according to Applied Biosystems’ (Applied Biosystems, Foster City, CA, USA) recommendations with RNaseP as the reference assay (reference gene) and transferred by hand to each row of a 96-well full-skirted plate.  10ul of each assay mix was then stamped into the appropriate reaction plate containing 10ng of dried down DNA per well.  The reaction plates were sealed with optical adhesive film, mixed on a plate vortex mixer, and centrifuged prior to running on the Applied Biosystems 7900HT Real Time PCR instrument. Thermal cycling was performed according to the manufacturer’s recommended protocol (Applied Biosystems.  Data were analyzed with SDS v2.4 software (Applied Biosystems).  The baseline was calculated automatically and the threshold was set manually based on the exponential phase of the amplification plot. Data were exported as a text file and imported into the Applied Biosystems CopyCaller v2.0 Program. Assays were analyzed by setting a negative control sample (selected from samples showing none of the CNVs under study by either PennCNV or CNAM) copy number to n=2 except for X chromosome assays, which were analyzed using n=1. For X chromosome CNVs both male and female control samples were used (3 male, 2 female). All other parameters were left as default.

**Pathway analysis.** Ninety of the genes analyzed were within CNV duplications and 63 genes were within CNV deletions. Eighty-seven genes were included since they were the gene nearest to a validated intergenic CNV. Gene abbreviations were batch converted to their Entrez Gene IDs using G:CONVERT [31,32]. Both DAVID and Ingenuity IPA use the right-tailed Fisher’s Exact test to calculate P-values representing the probability that selecting genes associated with that pathway or network is due to chance alone.

*Network Generation using IPA*: Each gene in our list of 240 was mapped to its corresponding object in Ingenuity’s Knowledge Base. These genes were overlaid onto a global molecular network developed from information contained in Ingenuity’s Knowledge Base. Networks then were algorithmically generated based on their connectivity. Both direct and indirect interactions were searched. Network scores are the –log P for the results of a right-tailed Fisher's Exact Test.

**Supplemental Results.**

**Principle component analysis (PCA).** Principal components analysis was used to assess the impact of population stratification within the study subjects. Principal components were calculated in SVS using default settings. All subjects were included in the calculation except those that failed data QC. Prior to calculating principal components, the SNPs were filtered so that only SNPs that met the following criteria were used: 1) autosomal SNPs only; 2) call rate > 0.95; 3) MAF > 0.05; 4) linkage disequilibrium R^2^ < 25% for all pairs of SNPs within a moving window of 50 SNPs. In total 2008 SNPs met these criteria. Self-reported ethnicity was used to group samples into “Caucasian” and “non-Caucasian” sets. A simple outlier detection algorithm was applied to stratify the subjects into the two groups. This was done by first calculating the Cartesian distance of each subject from the median centroid of the first two principal component vectors. After determining the third quartile (Q3) and inter-quartile range (IQR) of the distances, any subject with a distance exceeding Q3 + 1.5*IQR was determined to be outside of the main cluster, and therefore non-Caucasian. Five hundred sixty-four subjects were placed in the non-Caucasian category, including 207cases and 57 controls (Figure S1).

**
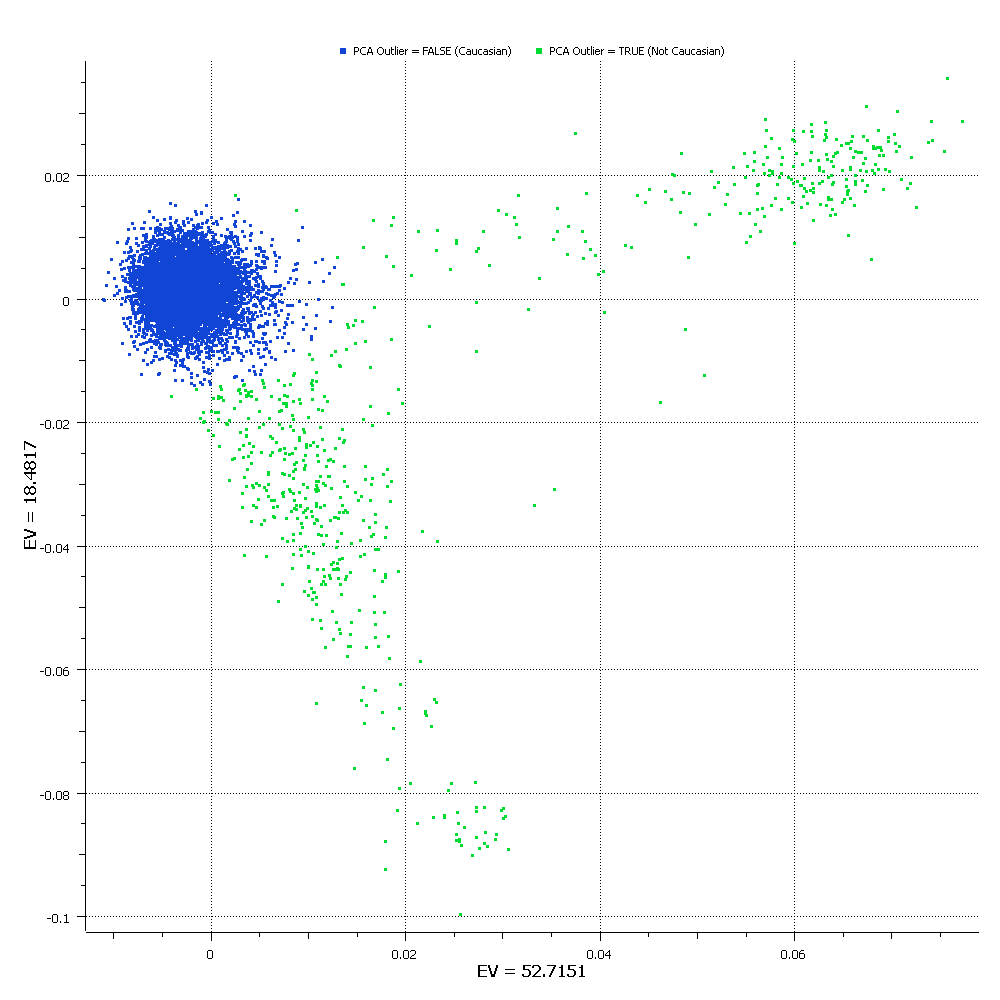
Figure S1: Plot of the first two principal components**, with plot symbols coded according to the estimated ethnicity of each subject. The axis labels indicate the eigenvalues for the principal components (EV=52.7151 is1^st^ PC, EV=18.4817 is 2^nd^ PC). Samples represented by blue dots are those included in the “Caucasian” group, while those represented by green dots were considered “non-Caucasian” and were excluded from all statistical analyses.

**Sample relatedness.** To insure that samples evaluated in this study were truly unrelated, SNP allele calls from 1,559 SNPs with call rates >0.99, MAF>0.05, and R^2^<10% for all pairs of SNPs within a moving window of 50 SNPs, were evaluated using the Identity by Descent Estimation tool in SVS (Golden Helix, Inc.). A small number of samples were removed due to duplicate enrollment in the study, but no other unexpected relationships were identified.
